# Harris Poll Migraine Report Card: population-based examination of high-frequency headache/migraine and acute medication overuse

**DOI:** 10.1186/s10194-024-01725-2

**Published:** 2024-02-26

**Authors:** Amaal J. Starling, Roger Cady, Dawn C. Buse, Meghan Buzby, Charlie Spinale, Kathy Steinberg, Kevin Lenaburg, Steven Kymes

**Affiliations:** 1 Mayo Clinic, Scottsdale, AZ USA; 2 RK Consults, Ozark, MO USA; 3 Missouri State University, Springfield, MO USA; 4 Axon-Therapeutics, San Diego, CA USA; 5 Albert Einstein College of Medicine, Bronx, NY USA; 6 Coalition for Headache and Migraine Patients (CHAMP), San Rafael, CA USA; 7 The Harris Poll, New York, NY USA; 8 Clusterbusters, Inc, Lombard, IL USA; 9 Lundbeck LLC, Deerfield, IL USA

**Keywords:** Acute medication overuse, High-frequency migraine, Chronic migraine, Patient perspective, Harris Poll, Survey

## Abstract

**Background:**

Migraine is a disabling neurologic disease that can fluctuate over time in severity, frequency, and acute medication use. Harris Poll Migraine Report Card was a US population-based survey to ascertain quantifiable distinctions amongst individuals with current versus previous high-frequency headache/migraine and acute medication overuse (HFM+AMO). The objective of this report is to compare self-reported experiences in the migraine journey of adults with HFM+AMO to those who previously experienced HFM+AMO but currently have a sustained reduction in headache/migraine frequency and acute medication use.

**Methods:**

An online survey was available to a general population panel of adults (≥18 years) with migraine per the ID Migraine™ screener. Respondents were classified into “current HFM+AMO” (within the last few months had ≥8 headache days/month and ≥10 days/month of acute medication use; *n*=440) or “previous HFM+AMO” (previously had HFM+AMO, but within the last few months had ≤7 headache days/month and ≤9 days/month of acute medication use; *n*=110). Survey questions pertained to demographics, diagnosis, living with migraine, healthcare provider (HCP) communication, and treatment.

**Results:**

Participants in the current HFM+AMO group had 15.2 monthly headache days and 17.4 days of monthly acute medication use in last few months compared to 4.2 and 4.1 days for the previous HFM+AMO group, respectively. Overall, current preventive pharmacologic treatment use was low (15-16%; *P*>0.1 for current vs previous) in both groups. Previous HFM+AMO respondents reported better current acute treatment optimization. More respondents with current (80%) than previous HFM+AMO (66%) expressed concern with their current health (*P*<0.05). More than one-third of both groups wished their HCP better understood their mental/emotional health (current 37%, previous 35%; *P*>0.1 for current vs previous) and 47% (current) to 54% (previous) of respondents worried about asking their HCP too many questions (*P*>0.1 for current vs previous).

**Conclusion:**

Apart from optimization of acute medication, medical interventions did not significantly differentiate between the current and previous HFM+AMO groups. Use of preventive pharmacological medication was low in both groups. Adults with current HFM+AMO more often had health concerns, yet both groups expressed concerns of disease burden. Optimization of acute and preventive medication and addressing mental/emotional health concerns of patients are areas where migraine care may impact outcomes regardless of their disease burden.

**Graphical Abstract:**

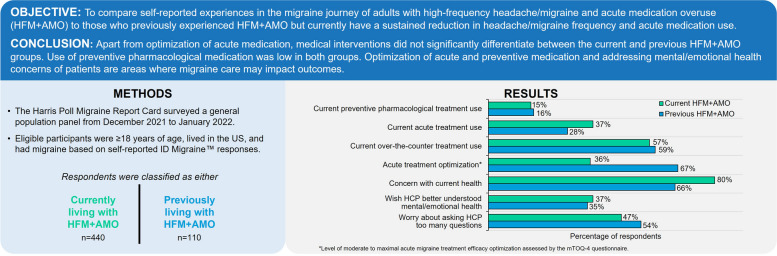

**Supplementary Information:**

The online version contains supplementary material available at 10.1186/s10194-024-01725-2.

## Introduction

Migraine, a common and disabling neurologic disease [1], is the second leading cause of global disability and the first among women aged 15-49 years [2]. According to US population-based studies, the prevalence of migraine is approximately 18% in women and 6% in men [3]. Migraine can negatively impact nearly all aspects of life, including work (absenteeism and presenteeism), school, family, finances, personal identity, and social interactions [4, 5]. Migraine is also associated with comorbidities, including cardiac, respiratory, pain, and psychiatric (such as depression and anxiety), among others [3, 6].

As migraine and headache frequency increases, so can acute headache/migraine medication (AHM) use. Poorly optimized acute medication (i.e., medication that is not efficacious, is poorly tolerated, or causes poor adherence), absent preventive medication among those who are candidates, and lack of non-medication strategies can exacerbate this problem. AHM can also have limited efficacy and/or limited sustained benefits, contributing to the need for more frequent dosing. In addition, AHM can be used/overused between attacks in an attempt to avoid an attack [7–10], all of which can potentially lead to acute medication overuse (AMO) [8] and medication-overuse headache (sometimes referred to as *medication adaptation headache* to lessen patient-related stigma [11]). AMO is defined as taking AHM either ≥10 days/month for most medications or ≥15 days/month for simple analgesics [12]. Adults with migraine and AMO often experience impairment in their day-to-day function and quality of life due to chronic symptoms that can worsen over time [7, 8].

Despite major scientific advancements in migraine treatment over the last 25 years, many aspects of the migraine experience, including care seeking and treatment, remain unchanged [13]. Migraine-associated disability rates remain high and appear to be increasing. Only half of adults with migraine seek consultation and obtain a diagnosis, and this rate has not improved. Use of preventive therapies remains very low (less than 20%) in relation to the rates of patients who meet criteria according to the American Headache Society 2021 consensus statement [3, 14–16]. Moreover, many adults with migraine continue to experience debilitating burden of disease at a substantial toll to individuals, families, and society [13].

This is the primary analysis of the Harris Poll Migraine Report Card (“Migraine Report Card”), a US population-based survey, designed to examine the migraine experience of adults currently experiencing high-frequency headache/migraine (HFM) and AMO versus those who previously experienced HFM+AMO. For this survey, HFM+AMO was defined as a diagnosis of migraine with a headache day frequency of ≥8 days/month and ≥10 days/month of AHM use within the last few months [17, 18]. Previous studies, including an American Migraine Prevalence and Prevention (AMPP) subgroup analysis [18], have suggested that the ≥15 headache days/month threshold is not the ideal number to differentiate between episodic and chronic migraine or the amount of burden one has [17]. These studies saw minimal difference in migraine burden between 8-14 headache days/month when compared to ≥15. Therefore, this distinction allowed exploring the challenges of not only those living / who have lived with chronic migraine (CM), but also those who have/had high-frequency episodic migraine (EM). Because of the burden associated with HFM+AMO, we hypothesized that those with current HFM+AMO would have a higher burden of disease and more negative impacts on their health-related quality of life than those with previous HFM+AMO and that this difference could provide guidance to healthcare providers. Given the self-reported, patient-directed nature of the survey, HFM+AMO is not synonymous with high-frequency EM, CM, or medication-overuse headache; however, respondents may have one or more of those diagnoses.

## Methods

### Survey design

This was a national, non-interventional, cross-sectional online survey fielded by The Harris Poll (a US-based market research and analytics company) and available to a general population panel from December 9, 2021, to January 10, 2022. The survey took ~15 minutes to complete and consisted of closed-ended questions. Respondents provided electronic informed consent prior to screening and were asked to read/agree to the Harris Poll privacy policy before continuing. This survey was not intended to provide clinical data for treatment decisions and was not conducted as a clinical trial; therefore, Institutional Review Board approval was not sought nor required. Survey respondents were compensated for their time/participation with loyalty points toward panel membership.

### Respondents

Respondents were recruited from online market research panels of members who agreed to participate in this type of research. Respondents had to be ≥18 years of age and live in the United States. Eligible survey respondents included those who screened positive for migraine based on self-reported ID Migraine™ responses [19]. ID Migraine™ is a validated 3-item screener that identifies individuals very likely to have migraine if they answer “yes” to 2 of the 3 items. The items ask whether headache has limited activities for ≥1 day within the past few months, whether nausea is experienced during headache, and whether there is light sensitivity during headache [19]. Respondents were classified as having either “current HFM+AMO” or “previous HFM+AMO.” Current HFM+AMO was defined as experiencing ≥8 days or parts of days/month with headache or migraine within the past few months and ≥10 days/month of any AHM use within the past few months. Previous HFM+AMO was defined as previously experiencing the thresholds for current HFM+AMO when their headache pattern was at its worst and now experiencing ≤7 days or parts of days/month with migraine within the past few months and ≤9 days/month of any AHM use within the past few months.

### Survey assessments

The Migraine Report Card (Supplement File 1) included screening questions to assess demographics (gender, age, race and ethnicity [presented in combined categories], and geographic location); overall health and comorbidities; and questions pertaining to migraine history, characteristics, and treatment. Treatments were those specific to headache/migraine care. Gender was captured as a single-choice question of “Are you…?” with response options of male, female, transgender, non-binary/gender non-conforming, or prefer not to answer. Questions pertaining to headache characteristics and treatment prompted respondents to: ‘Please assume that days with “headache” also refers to days with migraine and/or other types of headache.’ Therefore, data presented can relate to different types of headache diseases. The survey assessed the following self-reported experiences: diagnosis, healthcare provider (HCP) relationships and perceptions, symptom frequency and impact, disease burden and concerns, disease management goals, and perceptions of current/past acute and preventive treatments. In this survey, a respondent’s HCP refers to the primary provider that treated them for headaches/migraine.

Respondents who currently did/used/took something to treat migraine were prompted to answer the 6-item Migraine Treatment Optimization Questionnaire (mTOQ-6) [20]. A self-report questionnaire used to assess the optimization of acute migraine treatment. Each item is scored as never (1), rarely (2), less than half the time (3), and half the time or more (4). The mTOQ-6 total score (range of 6-24) is calculated by summing item scores, with higher scores indicating better acute treatment optimization [20]. The 4-item Migraine Treatment Optimization Questionnaire (mTOQ-4) is a validated subset of the mTOQ-6 used to assess treatment efficacy optimization, with optimization categorized as very poor (0), poor (1-5), moderate (6-7), and maximal (8) [21].

### Data analysis

No formal power calculations were conducted a priori. The sample size was determined based on the feasibility in the online panel and desire to balance and compare between the current and previous arms. The quota was set at *n*=400 (current HFM+AMO) and *n*=100 (previous HFM+AMO). The sample group used for weighting was the total sample of US age ≥18-years respondents, from which the quota groups in this study were selected subsets. Raw data were weighted via the Random Iterative Method (RIM) where necessary to align them with their actual proportions in the US population from benchmarks from the March 2021 US Census Bureau’s Current Population Survey Annual Socioeconomic Supplement^23^ by age (18+), education, sex, race, Hispanic ethnicity, US Census region, household income, household size, and marital status. Propensity score weighting was used to adjust for respondents’ propensity to be online.

RIM weighting uses an algorithm to put selected variables through an iterative process, adjusting each round and repeating until convergence is obtained when the targets for each variable have been met. In this survey, the data converged when the weighted distributions for each of the variables all matched the specified targets. This provided an even distribution of results across the dataset while balancing selected variables to the pre-determined targets. Specified variables were weighted simultaneously, but not in combination with one another. After RIM weighting, each respondent had a single weight value. These individual weight values were then capped based on standard parameters by sample size, to limit any extreme weighting or outliers. While unweighted sample sizes are presented, percentage values were calculated using weighted data. Because of this methodology, this survey’s specific migraine quota groups, current HFM+AMO or previous HFM+AMO, are representative of their respective overall populations within the US.

Descriptive statistics included mean and standard deviation for continuous variables and percentage rates for categorical variables. Statistically significant differences between the current and previous groups were determined by a standard, two tailed t-test of column proportions and means at the 90% (*P*<0.1) and 95% (*P*<0.05) confidence levels. The sampling precision of Harris online polls is measured by using a Bayesian credible interval. Prior information for this was a beta distribution with alpha parameter = 1 and beta parameter = 1, also colloquially known as the uninformed prior, which is the conjugate of prior likelihood. Likelihood distribution was a binomial distribution where each individual had a one or a zero depending on their response. These two distributions formed the posterior distribution known as beta binomial with alpha parameter equal to the number of respondents that gave a certain response plus one and a beta parameter of the number of respondents who did not give that response plus one. Because of the use of conjugate priors, no Metropolis Hastings or Gibbs sampling estimation was required. Statistical tests were only performed when sample size was ≥30. Sample data are accurate to within +5.3 percentage points using a 95% confidence level. This credible interval was wider among subsets of the surveyed population of interest. Data were analyzed using IBM® Quantum, version 5.8 (IBM Corporation., Armonk, NY, USA).

Respondents were required to answer each question (and any subquestions) before moving on in the survey, thus there were no missing data. However, questions of a sensitive nature included a prefer or decline to answer response. This percentage was relatively low across different questions.

## Results

### Survey population and demographics

A total of 550 US adults were categorized into the current HFM+AMO group (*n*=440; weighted *n*=493) or previous HFM+AMO (*n*=110; weighted *n*=57; Supplementary Figure 1). Representation of males and females was balanced in both groups, and racial demographics were generally representative of the US population (Table 1) and included considerable representation of Hispanic and Black (not Hispanic) adults.

**Table 1:** Respondent demographics and clinical characteristics

	**Current HFM+AMO (*n*=440)**	**Previous HFM+AMO (*n*=110)**
Age, mean (SD) years	41.1 (12.9)	47.2 (17.1)*
Gender, %		
Female	54	49
Male	44	49
Non-binary/Gender non-conforming	1	2
Transgender	1	0
Race/ethnicity, %^a^		
White, not Hispanic	57	75*
Hispanic	24*	13
Black or African American, not Hispanic	11	4
Asian, not Hispanic	2	3
Native American or Alaskan, not Hispanic	0	2*
More than one race	5	3
Age at first diagnosis, mean (SD)	23.9 (11.3)	25.0 (11.1)
Length of time since diagnosis, mean years (SD)	17.6 (12.0)	21.9 (15.3)*
Monthly headache days in last few months, mean (SD)	15.2 (5.80)*	4.2 (2.13)
Monthly acute medication use in last few months, mean (SD) days^b^	17.4 (6.4)*	4.1 (2.3)
Highest education level completed, %		
Less than high school	9	4
High school to less than 4-year college degree	57	59
4-year college degree or more	33	37
Has health insurance, %		
Yes	93	95
No	7	5
Total yearly household income, %		
Less than $15,000	7	8
$15,000 to $24,999	8	7
$25,000 to $34,999	9	5
$35,000 to $49,999	9	7
$50,000 to $74,999	19	16
$75,000 to $99,999	13	16
$100,000 or more	34	37

^*^Indicates significantly higher than the other group at the 95% confidence level (*P*<0.05).

^a^Participants were first asked: “Are you of Hispanic, Latino, or Spanish origin?” with response options of Yes or No. Then, participants were asked: “What is your race? Please select all that apply,” for which the following options were presented: White, Black or African American, Native American or Alaskan Native, Native Hawaiian or Pacific Islander, South Asian, Chinese, Korean, Japanese, Filipino, Arab/West Asian, Vietnamese, other Asian, and other race. Data were analyzed by separating Hispanic respondents from the race analysis

^b^Acute medication use is any over-the-counter or prescription medication to treat headaches. Percentages may not add up to 100% due to rounding

Abbreviations: HFM+AMO, high-frequency headache/migraine with acute medication overuse; SD, standard deviation

**Fig. 1:**
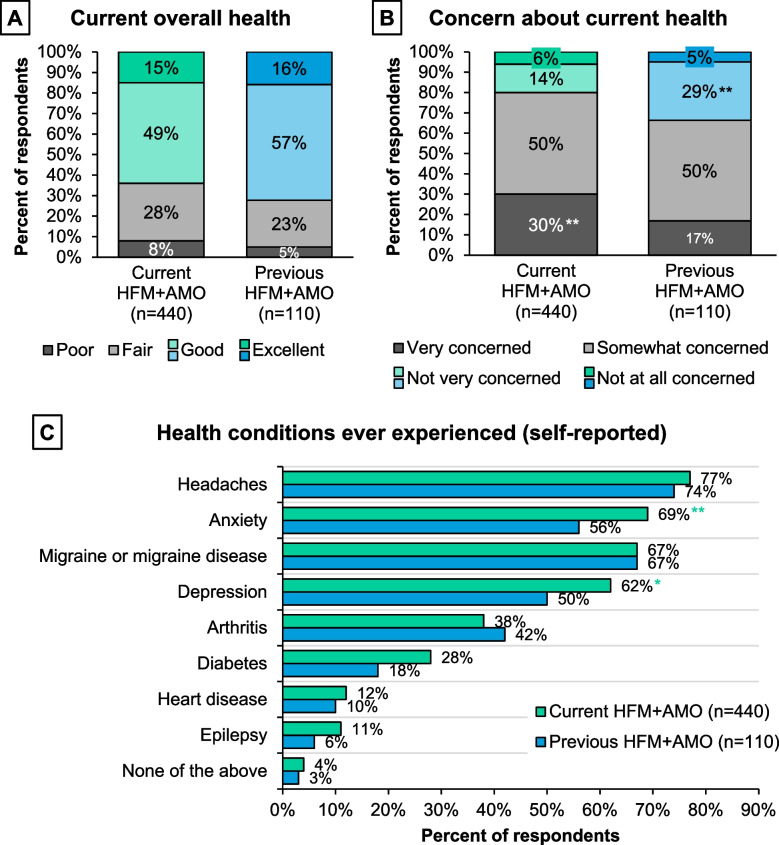
Current overall health (**A**), current health-related concern (**B**), and health issues ever experienced (**C**) for respondents with current or previous HFM+AMO. **A **All respondents were asked: “How would you describe your current overall health?” **B** All respondents were asked: “How concerned are you about your current overall health?” **C** All respondents were asked: “Have you ever experienced any of the following? Please select all that apply​”. *Indicates significantly higher than the other group at the 90% confidence level (*P*<0.1). **Indicates significantly higher than the other group at the 95% confidence level (*P*<0.05). Abbreviations: HFM+AMO, high-frequency headache/migraine with acute medication overuse

Respondents with previous HFM+AMO were an average of 6 years older than those with current (*P*<0.05). Most respondents in both groups were first diagnosed by an HCP with headache/migraine in their mid-20s (*P*>0.1; Table 1). Almost all (93-95%) respondents had some form of health insurance (*P*>0.1), which is consistent with the national percentage of 92% insured (as of August 2022). The most common self-reported diagnosis from a healthcare provider for respondents with previous HFM+AMO was migraine with/without aura (*P*>0.1), and for those with current, stress headache (*P*<0.05) (Supplemental Table 1). Most respondents with either current or previous HFM+AMO described their current overall health as “good” or “excellent” (Fig. 1A); despite this, many still expressed concern (*P*<0.05; Fig. 1B).

### Diagnosis

Ninety-five percent of respondents with current HFM+AMO and 92% with previous said they obtained a formal migraine or headache diagnosis from an HCP. Though all respondents met the screener algorithm for migraine via ID Migraine™, one-third of each group did not report ever experiencing migraine or migraine disease (*P*>0.1; Fig. 1C). Both groups self-reported having experienced comorbidities including depression, anxiety, arthritis, diabetes, heart disease, and epilepsy.

### Living with migraine

Both groups of respondents expressed some current headache/migraine concerns (i.e., that headaches might get worse, will damage their brain, or may impact their career/education). In the previous group, as headaches became less frequent, so did worries about family and finances (Supplemental Figure 2). Migraine also affected employment rates, with 34% of current HFM+AMO and 46% of previous not currently employed (*P*<0.1); of this unemployed group, 10% (current) and 7% (previous) were not employed or were unable to work due to a disability or illness and 7% (current) and 23% (previous) were retired.

Over 50% of respondents from both groups self-reported that headaches have a negative impact on overall quality of life (*P*>0.1 for current vs previous). Moreover, ≥50% of respondents with either current or previous HFM+AMO self-reported ever experiencing anxiety (*P*<0.05) or depression (*P*<0.1; Fig. 1C), and 48-56% said that headaches negatively impacted their mental/emotional health (*P*>0.1).

The top headache management goals for each group were preventing headaches (19% of each group; *P*>0.1), reducing the number of headache days (current 11%, previous 10%; *P*>0.1), reducing symptom severity (current 9%, previous 20%; *P*<0.05), eliminating or reducing pain during headache (current 10%, previous 6%; *P*>0.1), and having the freedom to live life (current 10%, previous 4%; *P*>0.1). Those with current or previous HFM+AMO who experienced helpful changes to improve their headaches made improving stress management a priority (current 45%, previous 48%; *P*>0.1) as well as limiting caffeine consumption and prioritizing healthy eating/drinking habits (current 57%, previous 54%; *P*>0.1).

### Treatment

**Fig. 2:**
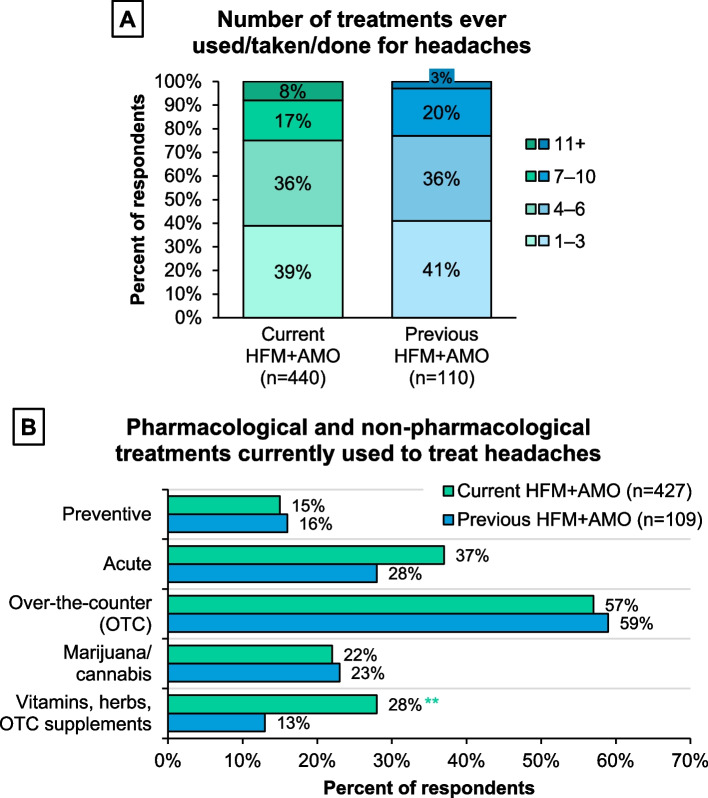
Treatment profile of respondents with current or previous HFM+AMO. **A** All respondents were asked: “Have you ever used, taken, or done any of the following to treat your headaches? Please select all that apply”. **B** Respondents who had ever used, taken, or done something to treat headache were asked: “Do you currently (or in the past few months) use, take, or do any of the following to treat your headaches? Please select all that apply.” OTC medications include acetaminophen and nonsteroidal anti-inflammatory drugs (NSAIDs). Acute medications include opioids, triptans, barbiturates, diclofenac, dihydroergotamine, gepants, ergotamines, and ditan. Preventive medications include anti-epileptic drugs (AEDs), Botox, and calcitonin gene-related peptide (CGRP) antagonists. **Indicates significantly higher than the other group at the 95% confidence level (*P*<0.05). Abbreviations: HFM+AMO, high-frequency headache/migraine with acute medication overuse

In general, respondents with either current or previous HFM+AMO felt their HCP’s overall knowledge of treatment options was high (Supplemental Figure 3). On average, respondents had used/taken approximately five pharmacologic (over-the-counter [OTC], acute, or preventive) or alternative treatments to treat headaches in their lifetime (Fig. 2A). Preventive pharmacologic treatment use at the time of survey was low, while prescription AHM and OTC medication use was higher (Fig. 2B). The previous group had more nonsteroidal anti-inflammatory drug (NSAID), triptan, and ergotamine use, whereas those with current HFM+AMO had higher use of gepants, diclofenac, and dihydroergotamine (*P*<0.05 or *P*<0.1 for the various medications; Supplemental Figure 4). Adults with current HFM+AMO were more likely to have currently or in the past few months used/taken/done non-medication therapies to treat their headaches (current 65%, previous 45%; *P*<0.05). The non-medication therapies included but were not limited to: vitamins or other OTC supplements; marijuana; massage, chiropractic, or biobehavioral therapies; or physical/occupational therapy.

**Fig. 3:**
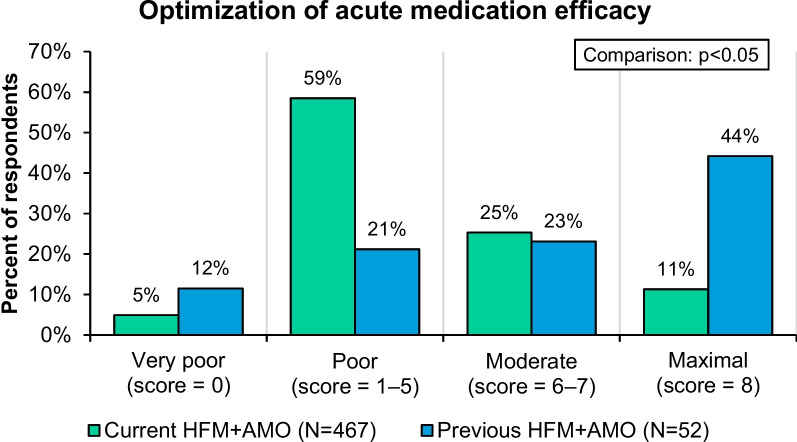
Level of acute migraine treatment efficacy optimization assessed by the mTOQ-4 questionnaire. Respondents who currently (or in the past few months) used, taken, or did something to treat headache were instructed: “Please answer the following questions about the medication(s) that you currently use to treat headaches”. Abbreviations: HFM+AMO, high-frequency headache/migraine with acute medication overuse; mTOQ-4, 4-item Migraine Treatment Optimization Questionnaire

The mTOQ-6 questionnaire revealed that respondents with previous HFM+AMO had higher overall acute treatment optimization than those with current HFM+AMO (mean [standard deviation] scores: previous, 20.2 [4.7]; current, 18.7 [3.6]; *P*<0.05). Categorical analysis of treatment efficacy optimization using mTOQ-4 scores showed that approximately twice as many current than previous HFM+AMO respondents had “poor” or “very poor” optimization and approximately four times as many previous than current HFM+AMO respondents had “maximal” optimization (*P*<0.05; Fig. 3). When examining individual mTOQ items, only 44% of current HFM+AMO respondents were comfortable enough half the time or more with their headache medication to be able to plan daily activities compared with 72% of the previous group (*P*<0.05; Supplemental Figure 5) and were more likely to want to change multiple aspects of their treatment (*P*<0.1 or *P*<0.05 for the different aspects). When asked what respondents would most like to change, if anything, about their current headache medication(s), this survey highlighted that duration (*P*<0.05), time to effect (*P*>0.1), and efficacy (*P*<0.05) were the top choices.

### HCP communication

**Fig. 4:**
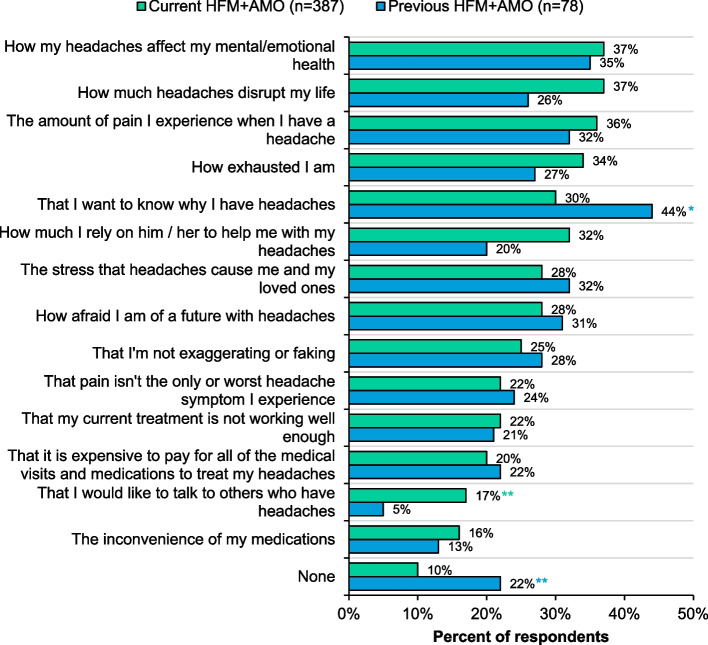
Healthcare needs that respondents with current or previous HFM+AMO wish their HCP, who currently manages their headaches, understood about their experiences living with headaches. Respondents currently seeing an HCP to manage their headaches were asked: “Which of the following, if any, do you wish your healthcare provider who currently manages your headaches, better understood about your experiences living with headaches? Please select all that apply”. *Indicates significantly higher than the other group at the 90% confidence level (*P*<0.1). Abbreviations: HCP, healthcare provider; HFM+AMO, high-frequency headache/migraine with acute medication overuse

Respondents were generally satisfied with their overall level of care. Those with previous HFM+AMO were nearly twice as likely to feel “somewhat” or “very” dissatisfied with their HCP (Supplemental Figure 3)—despite having fewer headache and AHM days—when compared to respondents with current HFM+AMO. Almost 1 in 5 respondents with current HFM+AMO said their HCP recommended a treatment plan without discussing it with them (*P*>0.1 for current vs previous). Additionally, 66% with current HFM+AMO wanted to talk more with their HCP about headache management goals compared to 43% with previous (*P*<0.05). More notably, respondents felt restricted when asking questions, with roughly half worried about being judged as a difficult patient if they ask too many questions (current 47%, previous 54%; *P*>0.1). Roughly one-third or more of respondents with current or previous HFM+AMO wished that their HCP better understood how headaches affect their mental health, better understood the amount of pain they experience when they have a headache, and knew why they had headaches (Fig. 4).

## Discussion

This US population-based survey focuses on the self-reported experiences of adults with HFM and high AHM use. This migraine “report card” provides a snapshot of migraine treatment and patient satisfaction among both current and previous HFM+AMO groups in light of current guidelines and treatment paradigms. This survey identified key areas where the burden of migraine affects adults with both current or previous HFM+AMO and areas for improvement in care, especially regarding treatment and HCP interactions. Within the Migraine Report Card survey results, we were surprised to find few distinguishing factors between the previous and current HFM+AMO groups relative to current overall health, headache-related impact on quality of life and mental/emotional health, and healthcare needs—indicating that, despite a sustained reduction in headache/migraine frequency and acute medication use, those previously experiencing HFM+AMO still experience substantial burden. These findings support previously published studies which have shown that significant burden can start at a headache/migraine frequency of 4 days/month [5, 18, 22].

Life with migraine has been explored and negative impacts reported in previous US population-based migraine surveys, including the American Migraine Prevalence and Prevention (AMPP, 2004-2009) [15], the Chronic Migraine Epidemiology and Outcomes (CaMEO, 2012-2013) [9], the Migraine in America Symptoms and Treatment (MAST, 2017-2018) [3], and the ObserVational survey of the Epidemiology, tReatment, and Care Of MigrainE (OVERCOME, 2018-2022) [23]. Here, as in those studies, we examined the challenges of living with migraine and patient satisfaction with treatment and HCP care.

The Migraine Report Card had good representation across gender, race, and ethnicity (Table 1), with higher representation of males (44-49%) when compared to CaMEO (~23%) [9], MAST (27%) [3], and OVERCOME (25.8%) [23], as well as those with CM (38-52%) when compared to AMPP (6.6%) [10], CaMEO (8.8%) [9], MAST (6.7%) [3], and OVERCOME (28.9%) [23]. The higher representation of males is significant and was intentional, as men are generally underrepresented in migraine-related studies; however, this did make the survey less representative of the US population with migraine by gender. The higher representation of individuals with CM allowed this survey to explicitly explore the burdens associated with HFM.

Similar to that reported from the AMPP study in 2007, in which 50% of people with migraine had severe impairment during their headaches [15], 15 years later we still see that over 50% of the current and previous respondents with HFM+AMO self-reported that migraine negatively affects their quality of life. These findings also align with CaMEO[9] and the American Registry for Migraine Research (ARMR) findings. Many barriers have prevented meaningful changes, including underdiagnosis, healthcare access, misdiagnosis, undertreatment, stigma, and poor HCP communication as well as factors that have not yet been uncovered [3, 23, 24].

Few specific elements of healthcare were observed to separate the outcomes of adults with current or previous HFM+AMO, highlighting that burden and disability can occur at various severities of HFM disease. For those with previous HFM+AMO, the burden of migraine may remain high due to their historical experiences and the fear of disease worsening in the future. Moreover, differential outcomes between groups could relate to the fact that the previous group was on average 6 years older than the current group and age-related disease improvement may be occurring. However, there is sparse research/evidence on the implications of a 6-year age difference on migraine remission rates. This age difference may also be an influencer on the difference in employment between the two groups. Additionally, the previous group was 75% White whereas the current group was more ethnically diverse (Table 1); more positive outcomes in the previous group could be related to healthcare access and utilization between different racial/ethnic groups [25, 26].

### Healthcare provider communication and care

Significantly, twice as many people in the previous HFM+AMO group compared to current were dissatisfied with the HCP who manages their migraine despite experiencing improvement in migraine frequency. The authors speculate that patients with severe disease who continue to have a high degree of satisfaction may have lower expectations of the HCP who manages their migraine. Moreover, 47-54% of respondents with current or previous HFM+AMO worry that if they ask too many questions, their HCP will think they are a difficult patient, suggesting that patient-perceived migraine-associated stigma is occurring. More than one-third of respondents wished their HCP better understood how headaches affect their mental/emotional health (Fig. 4). These findings are similar to AMPP, where respondents with high-frequency EM were 64% more likely to have depression and 57% more likely to have nervousness/anxiety [18]. Similar results were reported in MAST, where ~25% of participants reported moderate to severe anxiety/depression [3], and in CaMEO, where 56.6% and 48.4% of respondents with CM had experienced depression or anxiety, respectively [9]. Therefore, devoting extra time and resources to the mental/emotional health of patients with migraine may be beneficial.

Significantly, despite all respondents being highly likely to have migraine via ID Migraine™, one-third of each group did not self-identify as having migraine or migraine disease (Fig. 1C). Additionally, not all respondents self-reported a migraine diagnosis. The most common self-reported diagnosis for respondents with previous HFM+AMO was migraine with/without aura but for those with current it was stress headache, suggesting that migraine is underrecognized, underdiagnosed, and misdiagnosed. It is common for people with migraine to be misdiagnosed with diagnoses such as sinus or tension headache and/or mislabeled with terms such as stress headache. This confusion could impact seeking care in specialist settings as well as obtaining appropriate treatment options. This suggests that clinicians may need to better identify and diagnose migraine. Moreover, patient-centric educational programs or public migraine education campaigns can inform patients that the headaches and other accompanying symptoms they experience (nausea, sound/light sensitivity) are migraine, as well as provide guidance on optimization of acute medication and the prevention of medication overuse.

About one-third or more of respondents with current or previous HFM+AMO wished their HCP knew why they had headaches (Fig. 4), showing that patients want more information about their disease. Clinicians can make it a goal to explain migraine pathophysiology in patient-centered terms. This is an interesting finding and highlights a potential breakdown in HCP–patient communication. HCP migraine communication was highlighted in the American Migraine Communication Study from 2008 [27]. This study linked specific elements of HCP communication to clinical outcomes. As a future direction, evaluating existing patient education materials for potential gaps could help with this need identified by respondents.

### Treatment

The Migraine Report Card highlighted better optimization of current acute headache/migraine treatment(s) as a key difference between current and previous HFM+AMO. On average, current HFM+AMO respondents experienced 15 headache days/month and took AHM on 17 days/month. This compares to only 4 current headache and AHM days/month for previous HFM+AMO respondents. As shown through the mTOQ-6, most respondents with previous HFM+AMO had better overall current acute migraine medication optimization, and through the mTOQ-4, more respondents in the previous group had “maximal” optimization of usual acute treatment efficacy. Acute treatment optimization can be a preventive tool helping prevent chronification from episodic to chronic migraine. Adults with current HFM+AMO were more likely to have currently or in the past few months used, taken, or done non-medication therapies to treat their headaches despite likely paying substantial out-of-pocket expenses for these products, and potentially more than they would for prescribed medications, given the insurance status of the survey population. This finding may highlight decreased access to prescription medication or poor effectiveness of prescribed products.

The goal of migraine prevention is to reduce frequency and severity of migraine attacks and reliance on poorly tolerated or ineffective AHM. Per the 2021 AHS consensus statement on integrating new migraine treatments into clinical practice, preventive treatment should be offered to patients with either >6 migraine attacks/month, >4 with some disability, or >3 with severe disability and should be considered when a patient has either 4-5 headache days/month, 3 with some disability, or 2 with severe disability [16]. The consensus statement also notes that preventive treatment should be considered in patients with AMO [16]. Per the National Headache Foundation, prescribing migraine therapy should ultimately be determined by the clinician and patient and based on the specific circumstances and not determined solely by a one-size-fits-all, step-care model. In this survey, the current top goal for respondents with either current or previous HFM+AMO in managing headaches was prevention (preventing headaches, reducing the number of headache days, reducing symptom severity, and eliminating or reducing pain during headache), and—despite all respondents being candidates for migraine prevention when their migraine pattern was at its worst—at the time of this survey, only 15-16% of respondents were currently using a preventive medication and only 25-32% had ever used preventive medication. Similarly, AMPP found that 38.8% of participants were candidates for preventive treatment, but only 12.4% of them currently used preventive medication [15] and only 11.5% of respondents in the MAST study used preventive medication [3]. This demonstrates that preventives continue to be underutilized among adults who could benefit from them. There are several potential reasons why use was low, such as barriers to accessing care/speciality care (e.g., neurologists) or effective medications (due to step therapy/cost), under-counting of headache days (or headache-free days), or lack of clinicians encouraging preventive prescription use.

Optimization of acute and preventive treatment may be a critical factor in preventing disease progression from EM to CM, and clinicians should continually assess patients’ treatment optimization to ensure they receive effective care. Acute and preventive treatment optimization may also reduce the reliance on AHM to manage symptoms and the probability of acute medication overuse.

### Limitations

This survey has several limitations. All sample surveys and polls, whether they used probability sampling, are subject to other multiple sources of error which are most often not possible to quantify or estimate, including, but not limited to response bias, coverage error, error associated with nonresponse, error associated with question wording and response options, and post-survey weighting and adjustments. All data were collected via an online survey from a pool of people who agreed to participate in survey research. Therefore, respondents had to have internet access and all panelists had to complete a “confirmed” or “double opt-in” process to be included. Data were self-reported without supporting documentation or medical records for verification and may be subject to recall bias. Although the validated ID Migraine™ screener was used to positively screen respondents for migraine, it is not a diagnostic measure. Additionally, respondents were asked about the frequency of headache, which could include migraine or other headache types. In this survey ≥8 headache/migraine days was considered high frequency. While this number does not match with chronic migraine or high-frequency episodic migraine, ≥8 headache/migraine days is in alignment with other studies that have showed that there are small differences in burden between patients with 8-14 headaches days when compared to ≥15 headache days [17, 18]. In this survey, we defined AMO as a threshold of ≥10 days/month for all medication types for simplicity; however, per International Classification of Headache Disorders (3^rd^ edition) criteria, medication overuse is defined as ≥10 days/month (for more than 3 months) for ergotamine, triptans, opioids, and combination-analgesic medications and ≥15 days/month (for more than 3 months) for non-opioid analgesics, including acetaminophen, NSAIDs, and acetylsalicylic acid [12]. Thus, the label of HFM+AMO is slightly modified from ICHD-3 criteria. Also of note, in this survey, the respondent’s HCP refers to the primary provider that treated them for headaches/migraine; therefore, the type of provider may have varied per respondent (i.e., a primary care physician, allergist, headache specialist, neurologist, etc.). This may have affected the type of care each respondent received, particularly regarding preventive treatments, as these are mostly prescribed by neurologists and headache specialists. This survey was limited in the number of respondents (550 total); however, data were weighted to be representative of the larger population of US adults with migraine by age (18+), education, sex, race, Hispanic ethnicity, US Census region, household income, household size, and marital status. Additionally, the respondent population was highly insured, with 93-95% having some type of healthcare coverage; thus, we are unable to fully assess differences based on access to care/insurance, and this highly insured population may not be indicative of the overall US migraine population. The survey framed many questions as whether respondents had this symptom in the “last few months”; respondents may have responded based on their interpretation of this timeframe (e.g., 2-4 months). Despite these limitations, there are several strengths. Overall, there was great representation across males, females, and racial/ethnic groups, enabling this survey to be indicative of adults in the US population with current or previous HFM+AMO. This survey asked questions that addressed a specific subset of patients with migraine about life experiences, patient satisfaction with treatment and HCP care, and the stigma associated with migraine (to be reported in detail separately), which is lacking in some other US population-based migraine surveys.

## Conclusions

**Fig. 5:**
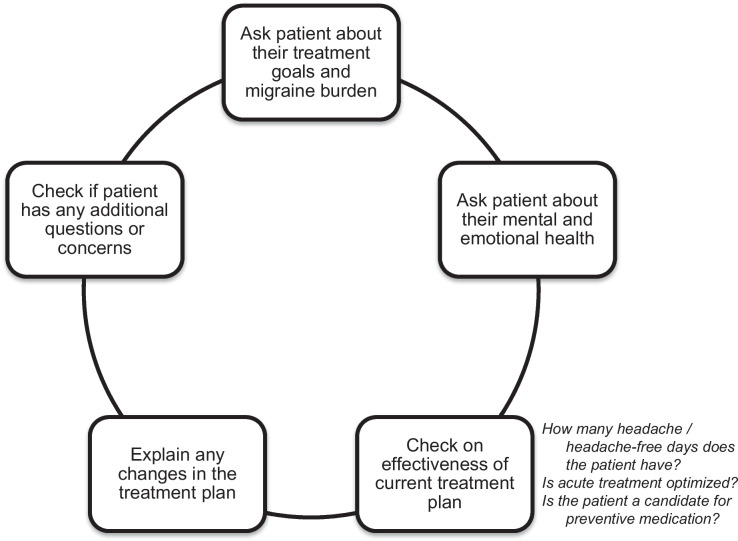
Respondent-identified key discussion points all healthcare providers treating patients with migraine should address during appointments

Despite new scientific advances in treatment, many people with migraine have acute medication overuse, significant disability, and are struggling to achieve their health goals. Respondents identified headache prevention as a top goal in migraine management, yet the conclusions from the Migraine Report Card survey mirrored results of other epidemiological studies conducted over the past 20 years showing low rates of preventive treatment use among those who are eligible and an overall lack of acute medication optimization. Moreover, respondents with migraine identified several key areas in which HCPs can provide better care, including improved communication about a patient’s migraine management goals, understanding a patient’s mental/emotional health and well-being (in addition to their disease state), and optimizing treatment response, which may be a critical factor in preventing the progression from EM to CM and breaking the cycle of increasing headache frequency and AHM use (Fig. 5) [3, 28, 29]. Overall, the results from this survey signify that there is a lot of room for improvement in the current standard of care for migraine. There is a need for better public health education and initiatives, further training in healthcare to identify and properly treat migraine/headache, and to continue to remove barriers to good care for patients with migraine.

### Supplementary Information

**Additional file 1:** **Supplemental Table 1.** Self-reported diagnosis from a healthcare professional. **Supplemental Figure 1.** Respondent flow diagram. **Supplemental Figure 2.** Current concerns about headaches for respondents with current or previous HFM+AMO. **Supplemental Figure 3.** Respondent overall satisfaction with specific attributes regarding their HCP who manages their headaches. (A) Attributes that patients are mostly satisfied with. (B) Attributes that patients are mostly dissatisfied with. **Supplemental Figure 4.** Medication(s) that respondents have ever used, taken, or done to treat headaches. **Supplemental Figure 5.** Respondent’s feelings toward medication(s) currently used to treat headaches assessed by the mTOQ-6 questionnaire.

**Additional file 2:** **Supplement File 1.** The Harris Poll Migraine Report Card survey.

## Data Availability

In accordance with EFPIA’s and PhRMA’s “Principles for Responsible Clinical Trial Data Sharing” guidelines, Lundbeck is committed to responsible sharing of clinical trial data in a manner that is consistent with safeguarding the privacy of patients, respecting the integrity of national regulatory systems, and protecting the intellectual property of the sponsor. The protection of intellectual property ensures continued research and innovation in the pharmaceutical industry. Deidentified data are available to those whose request has been reviewed and approved through an application submitted to https://www.lundbeck.com/global/our-science/clinical-data-sharing.

## Abbreviations

AMPP: American Migraine Prevalence and Prevention
AHM: Acute headache medication
AMO: Acute medication overuse
CaMEO: Chronic Migraine Epidemiology and Outcomes
CM: Chronic migraine
EM: Episodic migraine
HCP: Healthcare provider
HFM: Highfrequency headache/migraine
MAST: Migraine in America Symptoms and Treatment
Migraine Report Card: The Harris Poll Migraine Report Card
mTOQ-6: 6-item Migraine Treatment Optimization Questionnaire
NSAID: Nonsteroidal anti-inflammatory drug
OTC: Over-the-counter
OVERCOME: the ObserVational survey of the Epidemiology, tReatment, and Care Of MigrainE
