# Supplementary material for: Harris Poll Migraine Report Card: population-based examination of high-frequency headache/migraine and acute medication overuse
Source: J Headache Pain. 2024 Feb 26;25(1):26. doi: 10.1186/s10194-024-01725-2 (PMC10895775; doi:10.1186/s10194-024-01725-2)
Supplement: Supplementary file 1 — Additional file 1: Supplemental Table 1. Self-reported diagnosis from a healthcare professional. Supplemental Figure 1. Respondent flow diagram. Supplemental Figure 2. Current concerns about headaches for respondents with current or previous HFM+AMO. Supplemental Figure 3. Respondent overall satisfaction with specific attributes regarding their HCP who manages their headaches. (A) Attributes that patients are mostly satisfied with. (B) Attributes that patients are mostly dissatisfied with. Supplemental Figure 4. Medication(s) that respondents have ever used, taken, or done to treat headaches. Supplemental Figure 5. Respondent’s feelings toward medication(s) currently used to treat headaches assessed by the mTOQ-6 questionnaire. [file 10194_2024_1725_MOESM1_ESM.pdf]

Supplemental Materials

Supplemental Figures

Supplemental Figure 1. Respondent flow diagram

Supplemental Figure 2. Current concerns about headaches for respondents with current or previous HFM+AMO

Supplemental Figure 3. Respondent overall satisfaction with specific attributes regarding their HCP who manages their headaches. (A) Attributes that patients are mostly satisfied with. (B) Attributes that patients are mostly dissatisfied with

Supplemental Figure 4. Medication(s) that respondents have ever used, taken, or done to treat headaches

Supplemental Figure 5. Respondent’s feelings toward medication(s) currently used to treat headaches assessed by the mTOQ-6 questionnaire

Supplemental Figure 1. Respondent flow diagram

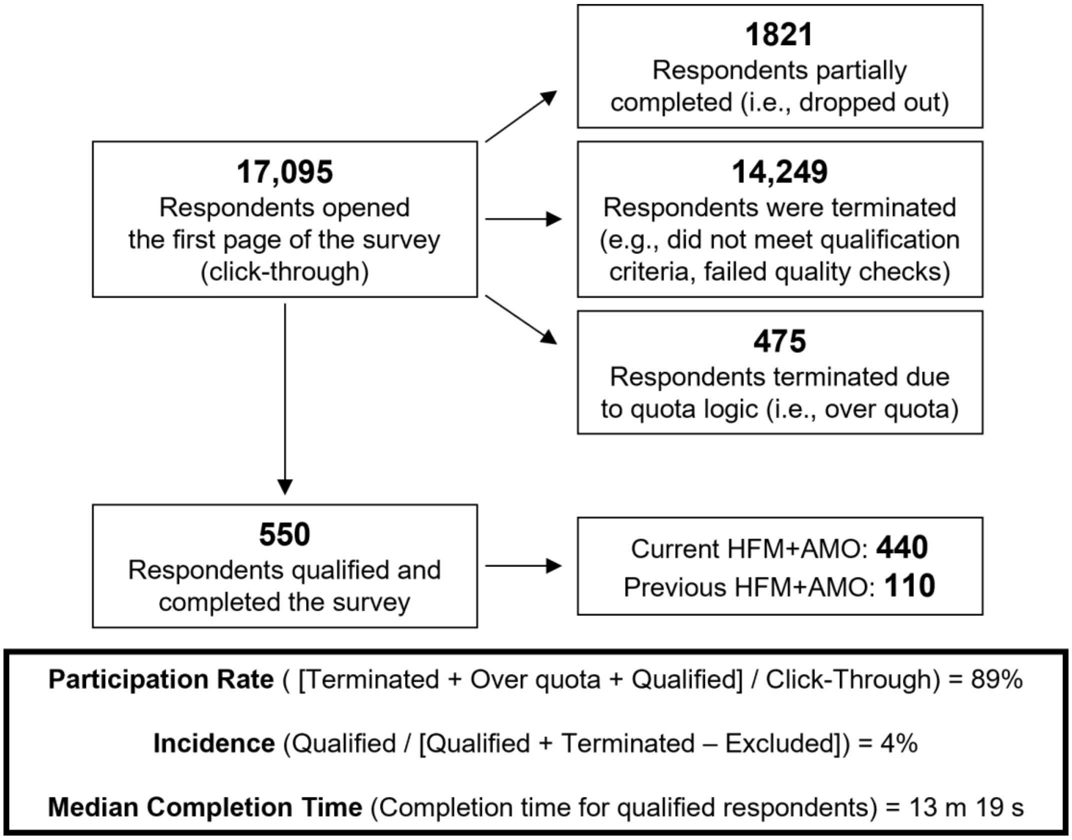

Abbreviations: HFM+AMO, high-frequency frequency headache/migraine with acute medication overuse.

Supplemental Figure 2. Current concerns about headaches for respondents with current or previous HFM+AMO

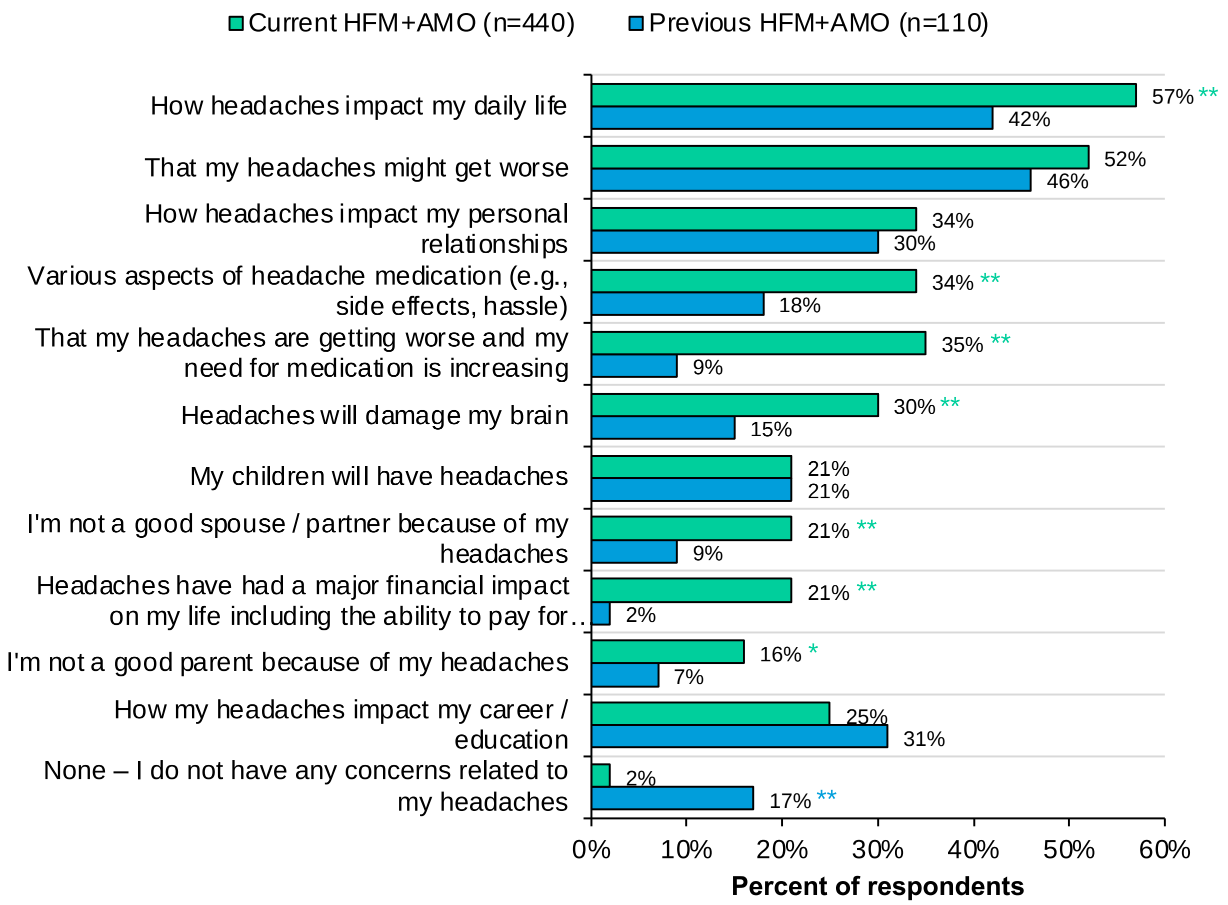

All respondents were asked: "Which of the following are you currently concerned about related to your headaches? Please select all that apply."

*Indicates significantly higher than the other group at the 90% confidence level (P<0.1). **Indicates significantly higher than the other group at the 95% confidence level (P<0.05).

Abbreviations: HFM+AMO, high-frequency headache/migraine with acute medication overuse.

Supplemental Figure 3. Respondent overall satisfaction with specific attributes regarding their HCP who manages their headaches. (A) Percentage of patients mostly satisfied with attributes. (B) Percentage of patients mostly dissatisfied with attributes

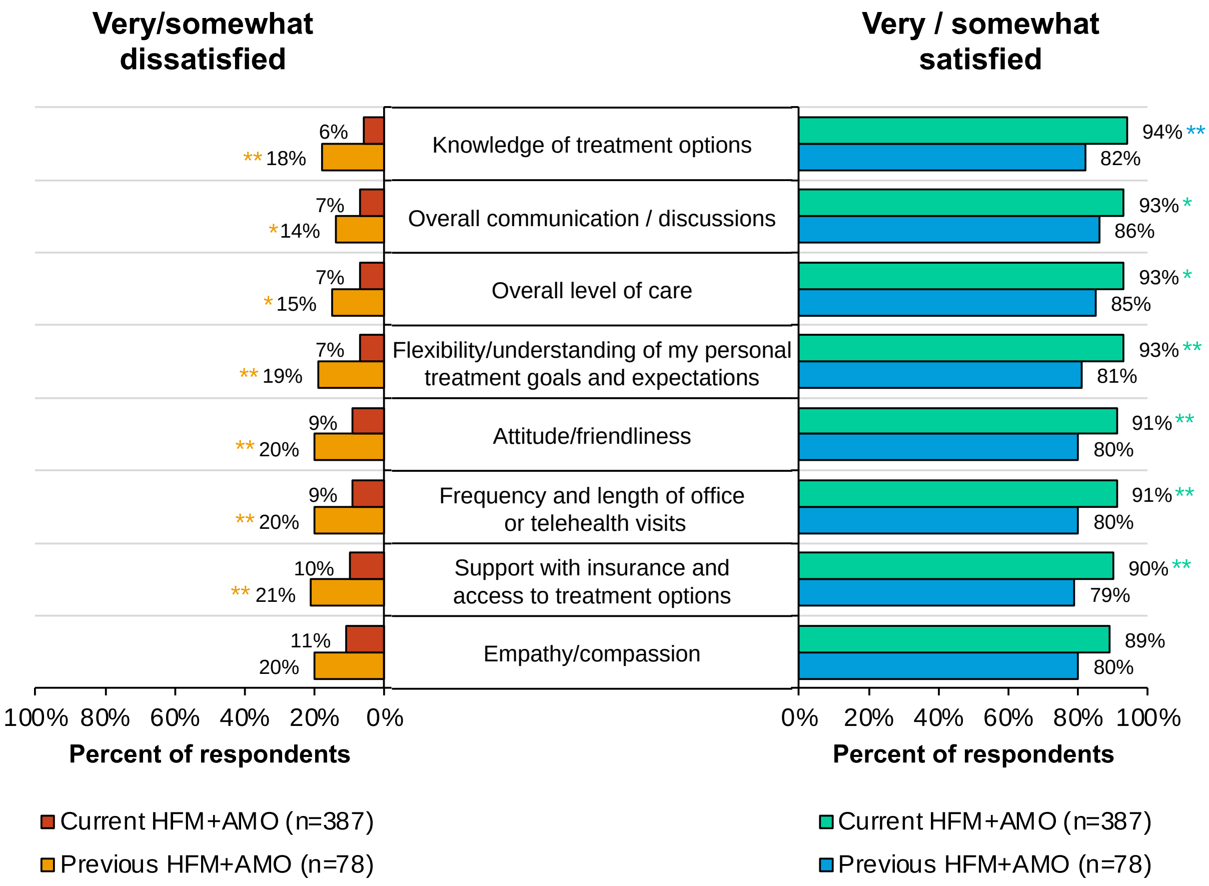

Respondents currently seeing an HCP to manage their headaches were asked: “Thinking about your healthcare provider who currently manages your headaches, how satisfied are you with each of the following?" Possible responses: very dissatisfied, somewhat dissatisfied, somewhat satisfied, very satisfied.

*Indicates significantly higher than the other group at the 90% confidence level (P<0.1). **Indicates significantly higher than the other group at the 95% confidence level (P<0.05).

Abbreviations: HCP, healthcare provider; HFM+AMO, high-frequency headache/migraine with acute medication overuse.

Supplemental Figure 4. Medication(s) that respondents have ever used, taken, or done to treat headaches

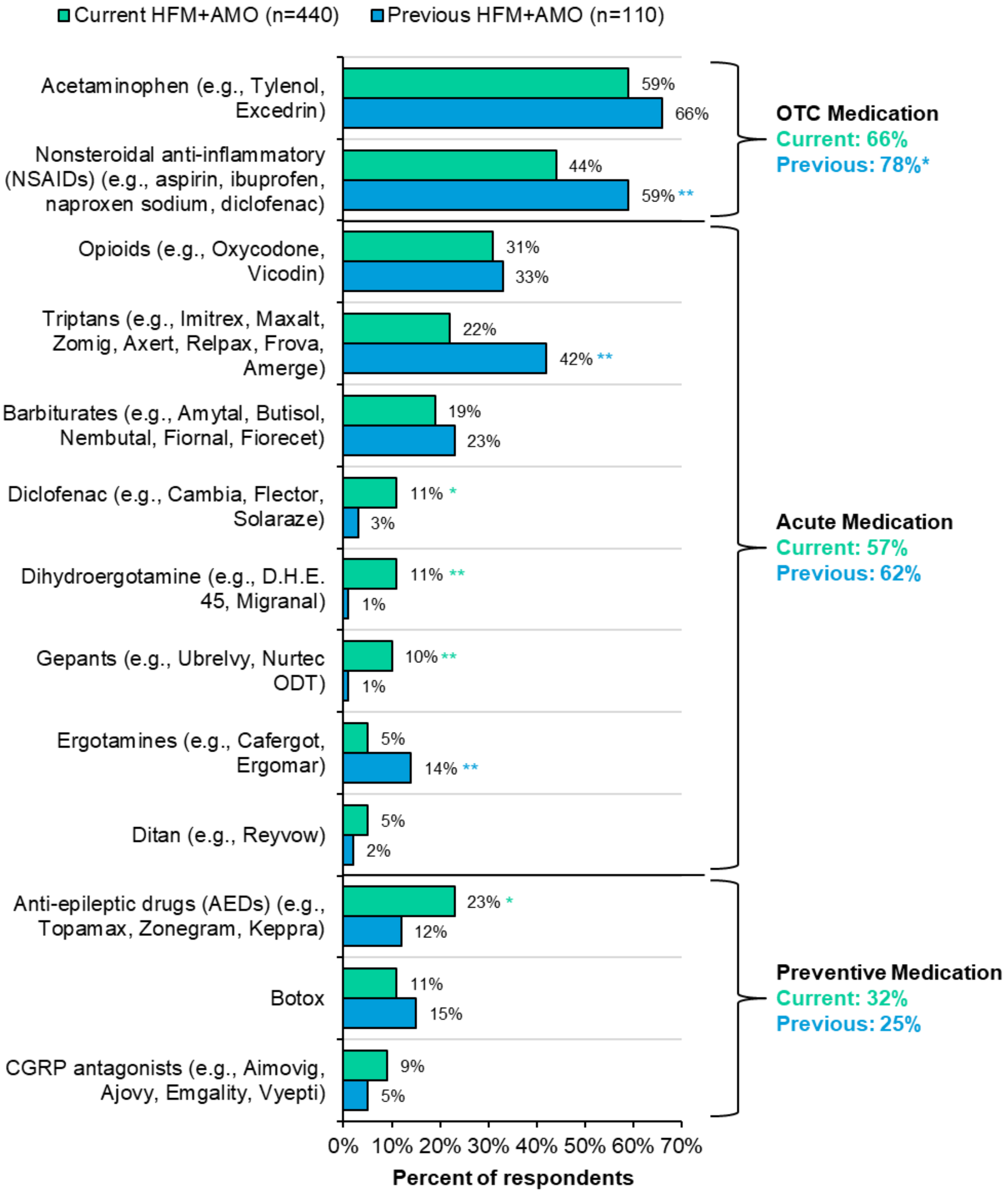

All respondents were asked: "Have you ever used, taken, or done any of the following to treat your headaches? Please select all that apply."

*Indicates significantly higher than the other group at the 90% confidence level (P<0.1). **Indicates significantly higher than the other group at the 95% confidence level (P<0.05).

Abbreviations: CGRP, calcitonin gene-related peptide; HFM+AMO, high-frequency headache/migraine with acute medication overuse; NSAIDs, nonsteroidal anti-inflammatory drugs; OTC, over-the-counter.

Supplemental Figure 5. Respondent's feelings toward medication(s) currently used to treat headaches assessed by the mTOQ-6 questionnaire

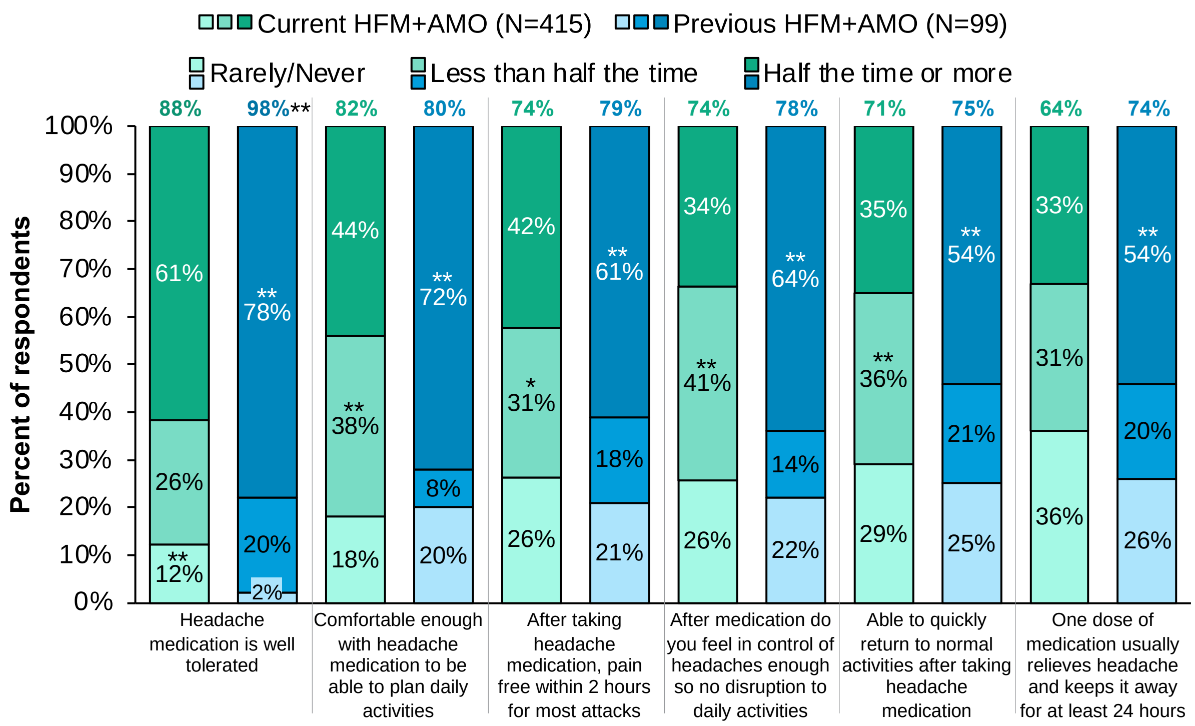

Respondents who currently (or in the past few months) used, taken, or did something to treat headache were asked: "Please answer the following questions about the medication(s) that you currently use to treat headaches."

Top row of numbers is the sum of "half the time or more" and "less than half the time."

*Indicates significantly higher than the other group at the 90% confidence level (P<0.1). **Indicates significantly higher than the other group at the 95% confidence level (P<0.05).

Abbreviations: HFM+AMO, high-frequency headache/migraine with acute medication overuse; mTOQ-6, 6-item Migraine Treatment Optimization Questionnaire.
