# Supplementary material for: Harris Poll Migraine Report Card: population-based examination of high-frequency headache/migraine and acute medication overuse
Source: J Headache Pain. 2024 Feb 26;25(1):26. doi: 10.1186/s10194-024-01725-2 (PMC10895775; doi:10.1186/s10194-024-01725-2)
Supplement: Supplementary file 2 — Additional file 2: Supplement File 1. The Harris Poll Migraine Report Card survey. [file 10194_2024_1725_MOESM2_ESM.pdf]

Harris Poll Migraine Report Card Survey

Thank you for agreeing to take this survey. Our first few questions are for classification purposes and will help us determine which questions to ask you later; they will also help us properly analyze responses. As you may know, we never disclose the identity of any individual and your answers will always be kept strictly confidential. We report results only for groups of people, not for individuals.

During the survey, please do not use your browser's FORWARD and BACK buttons. Instead, please always use the button below to move through the survey. Please be aware that once you've answered a question, you might not be able to go back and change your answer.

The progress bar below indicates approximately what portion of the survey you have completed.

Simply click on the button at the bottom of the page to begin the survey.

SCREENING QUESTIONS

Thank you for agreeing to participate in this survey. Your views are important to us and your answers will be kept in strict confidence. Please click here to read our privacy policy before agreeing to continue with the survey.

- I agree to continue
- I do not agree

In which country or region do you currently reside?

- United States of America
- Australia
- Brazil
- Canada
- China
- France
- Germany
- India
- Italy
- Japan
- Mexico
- Russian Federation
- Spain
- United Kingdom
- Other country

Are you…?

- Male
- Female
- Transgender
- Non-binary or gender non-conforming
- Prefer not to answer

What is your age? [RANGE 0 -120]

|__|__|__|

In what state or territory do you currently reside?

- Alabama
- Alaska
- Arizona
- Arkansas
- California
- Colorado
- Connecticut
- Delaware
- District of Columbia
- Florida
- Georgia
- Hawaii
- Idaho
- Illinois
- Indiana
- Iowa
- Kansas
- Kentucky
- Louisiana
- Maine
- Maryland
- Massachusetts
- Michigan
- Minnesota
- Mississippi
- Missouri
- Montana
- Nebraska
- Nevada
- New Hampshire
- New Jersey
- New Mexico
- New York
- North Carolina
- North Dakota
- Ohio
- Oklahoma
- Oregon
- Pennsylvania
- Rhode Island
- South Carolina
- South Dakota
- Tennessee
- Texas
- Utah
- Vermont
- Virginia
- Washington
- West Virginia
- Wisconsin
- Wyoming
- American Samoa
- Federated States of Micronesia
- Guam
- Marshall Islands
- Northern Mariana Islands
- Palau
- Puerto Rico
- Virgin Island

What is your zip code?

|__|__|__|__|__|

Are you of Hispanic, Latino, or Spanish origin?

- Yes
- No

What is your race? Please select all that apply.

- White
- Black or African American
- Native American or Alaskan Native
- South Asian
- Chinese
- Korean
- Japanese
- Filipino
- Arab/West Asian
- Vietnamese
- Other Asian
- Native Hawaiian or Pacific Islander
- Other race

How would you describe your current overall health?

- Poor
- Fair
- Good
- Excellent

How concerned are you about your current overall health?

- Not at all concerned
- Not very concerned
- Somewhat concerned
- Very concerned

Have you ever experienced any of the following? Please select all that apply.

- Anxiety
- Arthritis
- Epilepsy
- Heart disease
- Migraine or migraine disease
- Headaches
- Depression
- Diabetes
- None of the above
- Decline to answer

Please indicate if you <u>currently or within the last few months</u> experience(d) any of the following with your headaches and/or migraines. Please select all that apply.

- Limited your ability to work, study, or do what you needed to do
- You felt nauseated or sick to your stomach
- Light bothered you (a lot more than when you don't have a headache)
- None of the above

At a time in your life when your <u>headache pattern was at its worst</u>, please indicate if you ever experienced any of the following with your headaches and/or migraines. Please select all that apply.

- They limited your ability to work, study, or do what you needed to do
- You felt nauseated or sick to your stomach
- Light bothered you (a lot more than when you didn't have a headache)
- None of the above

<u>Thinking about the last few months</u>, how many days per month have you experienced a headache? Please include ALL days with headache pain of any kind, including migraine, lasting more than 30 minutes.

|__|__| days per month [RANGE = 0–30]

<u>Thinking about when your headache pattern was at its worst</u>, how many days per month did you experience a headache? Please include ALL days with headache pain of any kind, including migraine, lasting more than 30 minutes.

- |__|__| days per month [RANGE = 0–30]
- NA – my headache pattern is currently at its worst

<u>Thinking about the last few months</u>, how many days per month did you take any over-the-counter <u>or</u> prescription medication to treat your headache attack?

|__|__| days per month [RANGE = 0–30]

<u>Thinking about when your headache pattern was at its worst</u>, how many days per month did you take an over-the-counter <u>or</u> prescription medication to treat your headache attack?

- |__|__| days per month [RANGE = 0–30]
- NA – my headache pattern is currently at its worst

At this point, quality respondents were classified into current and previous groups.

Group 1: **Previous** self-reported high frequency headache days and high frequency medication use
- US residents
- Aged 18+
- Had migraine
- Historical frequency of $\geq$8 days/parts of day with headache or migraine per month and any acute headache medication use $\geq$10 days/month when their headache pattern was at its worst
- Now $\leq$9 days/month of any acute headache medication use and $\leq$7 days/parts of days with migraine per month in the past 3 months

Group 2: **Current** self-reported high frequency headache days and high frequency medication use
- US residents
- Aged 18+
- Has migraine
- Frequency of ≥8 days/parts of day with headache or migraine per month and ≥10 days/month of any acute headache medication use in past 3 months

<u>MAIN SURVEY</u>: DIAGNOSIS/PROFILE

How old were you when you experienced what you believe was your first migraine? Your best estimate is fine.

- |__|__| years old
- I have never had a migraine

For the following questions, please assume that days with "headache" also refers to days with migraine and/or other types of headache.

Have you <u>ever</u> received a diagnosis from a healthcare professional for any of the following types of migraine or headache? Please select all that apply.

- Migraine with or without aura
- Chronic migraine
- Menstrual migraine
- Other type of migraine
- Sinus headache
- Stress headache
- Tension headache
- Post-traumatic headache (headache due to concussion or traumatic brain injury)
- Cluster headache
- Other type of headache
- None – I have not been formally diagnosed by a healthcare professional with a migraine/headache condition

How old were you when you were first <u>diagnosed</u> with any migraine or headache by a healthcare professional? If you have received multiple diagnoses, please think of your first diagnosis. Your best estimate is fine.

|__|__| years old

Which healthcare professional(s), if any, have you seen to manage your headaches? Please include <u>all</u> healthcare professionals who are helping/have helped you diagnose your headache, treat your headache symptoms, manage your medication, treat other headache symptoms, etc.

- Primary care physician (PCP)/ family physician, general practitioner/internist
- Neurologist
- Headache specialist
- Nurse practitioner/physician assistant
- Psychiatrist/psychologist/ therapist
- Holistic practitioner (e.g., naturopath/herbalist/ homeopath)
- Doctor of osteopathic medicine
- Ophthalmologist
- Gynecologist/ObGyn
- Pain specialist
- Physical therapist/occupational therapist/chiropractor
- Dentist/orthodontist
- Otolaryngologist or ear/nose/throat specialist (ENT)
- Allergist or immunologist
- Physiatrist (Physical medicine and rehabilitation)
- Other
- None

Which of the following is the main healthcare professional currently helping you manage your headaches?

- Primary care physician (PCP)/ family physician, general practitioner/Internist
- Neurologist
- Headache specialist
- Nurse practitioner/physician assistant
- Psychiatrist/psychologist/ therapist
- Holistic practitioner (e.g., naturopath/herbalist/ homeopath)
- Doctor of osteopathic medicine
- Ophthalmologist
- Gynecologist/ObGyn
- Pain specialist
- Physical therapist/occupational therapist/chiropractor
- Dentist/orthodontist
- Otolaryngologist or ear/nose/throat specialist (ENT)
- Allergist or immunologist
- Physiatrist (Physical medicine and rehabilitation)
- Other
- None

When was the last time you saw the main healthcare professional helping you manage your headaches?

- Less than 3 months ago
- 3 to 6 months ago
- 7 to 11 months ago
- 1 to 2 years ago
- 3 to 4 years ago
- 5 or more years ago

MAIN SURVEY: LIVING WITH MIGRAINE

For the following questions, please assume that days with "headache" also refers to days with migraine and/or other types of headache.

Which of the following headache-associated symptoms, if any, do you find to be the most bothersome, other than headache or head pain? Please select one.

- Mood changes
- Sensitivity to light
- Sensitivity to smell
- Sensitivity to sound
- Speech difficulty
- Pressure/tightness
- Sleep disturbance
- Visual impact
- Aura (e.g., flashing lights, intense head pain, zigzag lines)
- Dizziness
- Allodynia (e.g., skin sensitivity when wearing a ponytail, shaving face hurts)
- Pain exacerbation with activity
- Pain
- Anatomical (bodily) pain
- Eye pain
- Neck pain
- Nausea/vomiting
- Throbbing/pulsation pain
- Sensory disturbance (e.g., tingling in hands or face, vision changes)
- Cognitive disruption (e.g., memory problems, difficult concentrating, feeling fuzzy headed)
- Fatigue
- Inactivity
- Other
- None
- N/A - I do not experience any headache symptoms

Which of the following changes, if any, have been helpful at improving your headaches? Please select all that apply.

- Made sleep a priority and/or improved my sleep
- Made exercise a priority and/or improved my exercise and movement
- Made healthy eating and drinking habits a priority and/or improved my diet and nutrition
- Limited caffeine consumption or stopped altogether
- Kept a headache diary or calendar
- Stopped smoking (e.g., tobacco, marijuana)
- Participated in one or more behavioral therapies for migraine/headache (e.g., cognitive behavioral therapy, biofeedback, relaxation therapies, mindfulness-based therapies, acceptance and commitment therapy)
- Made managing stress a priority and/or improved my stress management
- Other
- None

Please indicate how often (never, rarely, sometimes, often, always) you experience the following as a result of your headaches.

- Some people acted as though it was my fault I have this illness (migraine/headache)
- Because of my illness, I felt left out of things
- Because of my illness, people were unkind to me
- I felt embarrassed about my illness
- I felt embarrassed because of my physical limitations
- Because of my illness, some people seemed uncomfortable with me
- Because of my illness, some people avoided me
- Because of my illness, people avoided looking at me

Do your headaches have a <u>negative impact</u> on any of the following aspects of your life? Please select all that apply.

- My overall quality of life
- My finances
- My ability to work or how well I perform at work
- My ability to go to school or how well I perform in school
- My mental/emotional health
- My relationship with my spouse or significant other
- My relationship(s) with my child(ren)
- My relationships with other family members and/or friends
- My ability to participate in the hobbies and activities I enjoy
- My ability to take care of myself
- My ability to take care of others
- My self confidence
- My ability to go on a business or work trip
- My vacations
- My ability to plan future activities
- My ability to play and have fun
- Other
- None

Currently, what is your <u>top</u> goal for managing your headaches? Please select one.

- Reduce the level of worry or anxiety I experience about the next headache
- Increase my level of physical activity
- Substantially reduce or get rid of my pain during headaches
- Reduce the severity of my headaches or my other symptoms (beyond head pain) during headaches
- Simplify my treatment plan
- Reduce my need for urgent care/emergency department visits
- Reduce my need for acute medication
- Prevent headaches/ have no or fewer headaches
- Shorten the length of my headaches
- Reduce the number of headache days I experience per month
- Start or get back to work/school and be more productive and perform better
- Reduce the negative impact of headaches on my relationships with my friends and family
- Reduce or eliminate my most bothersome headache symptom(s)
- Have the freedom to live my life
- Think more clearly
- Other
- I have no goals for managing migraine

Which of the following are you <u>currently</u> concerned about related to your headaches? Please select all that apply.

- How headaches impact my daily life
- That my headaches might get worse
- That my headaches are getting worse and my need for medication is increasing
- Various aspects of headache medication (e.g., side effects, hassle)
- Headaches have had a major financial impact on my life including the ability to pay for medication
- How headaches impact my personal relationships
- How my headaches impact my career/education
- Headaches will damage my brain
- I’m not a good parent because of my headaches
- I’m not a good spouse/ partner because of my headaches
- My children will have headaches
- Other
- None – I do not have any concerns related to my headaches

Please indicate yes or no for each of the following.

- A family member of mine is/was formally diagnosed by a healthcare professional with migraine
- A family member of mine has/had migraine
- I have experienced one or more traumatic brain injuries or concussions in my life
- I currently use tobacco on a regular basis (i.e., weekly, or monthly)
- I used to use tobacco on a regular basis (i.e., weekly, or monthly)
- I am a regular user of caffeine

<u>MAIN SURVEY</u>: HCP COMMUNICATION

For the following questions, please assume that days with "headache" also refers to days with migraine and/or other types of headache.

Which of the following, if any, <u>best</u> describes the role of your healthcare provider who currently manages your headaches?

- My healthcare professional recommends a treatment plan for me without involving me in a discussion
- My healthcare professional involves me in a discussion and considers my preferences and concerns when determining the course of treatment
- My healthcare professional and I make decisions together about my treatment
- I request specific treatments from my healthcare professional, and they consider my requests when determining treatment
- None of the above

Approximately how often do you typically visit your healthcare provider who currently manages your headaches? Please include both in-person and telehealth appointments.

- Once a month or more frequently
- Once every 2 to 3 months
- Once every 4 to 6 months
- Once every 7 to 11 months
- Once a year of less often

Which of the following, if any, do you discuss <u>at the majority of your visits</u> with your healthcare provider who currently manages your headaches? Please select all that apply.

- How my current medication may help or hurt my treatment goals
- All my prescription medication options, including effectiveness and side effects
- Taking over-the-counter medications (e.g., ibuprofen)
- The potential benefits of migraine prevention medication
- How much and when to take/use medication during a headache
- Whether there has been a change in my headaches since my last visit
- Nonpharmacologic therapies (e.g., neurostimulation, behavioral therapy, alternative/complementary therapy)
- The mental/emotional health impacts (e.g., depression, anxiety) of headaches
- How I can get more resources and information about my condition (e.g., assistance programs, patient organizations, support groups)
- Access to treatments based on my insurance coverage
- My quality of life
- Adjustments to my lifestyle to improve my headache pattern
- How headaches are impacting or interfering in my life
- How well my current medication is working including any side effects
- My treatment goals
- Other
- None

Thinking about your healthcare provider who currently manages your headaches, how <u>satisfied</u> are you with each of the following? Possible responses: very dissatisfied, somewhat dissatisfied, somewhat satisfied, very satisfied.

- Overall communication/ discussions
- Overall level of care
- Frequency and length of office or telehealth visits
- Knowledge of treatment options
- Support with insurance and access to treatment options
- Attitude/friendliness
- Flexibility/understanding of my personal treatment goals and expectations
- Empathy/compassion

How <u>satisfied</u> are you with how your healthcare provider who currently manages your headaches communicates with you about each of the following aspects of headache management? Possible responses: very dissatisfied, somewhat dissatisfied, somewhat satisfied, very satisfied.

- All of my medication options, including their effectiveness and side effects
- All of my non-medication options, including alternative/complementary therapies, for managing my headaches
- The potential benefits of migraine prevention medication
- How much/what type of medication I should take during a headache
- Whether my headaches are getting worse
- The mental/emotional health impacts of headaches
- How my medication type (i.e., injection, infusion, oral) and/or frequency (e.g., daily, weekly, monthly) impacts my quality of life
- How I can get more resources and information about my condition (e.g., assistance programs, patient organizations, support groups)
- How things are going at home and work

With respect to your communication with your healthcare provider who currently manages your headaches, how much do you agree or disagree with each of the following statements? Possible responses: strongly disagree, somewhat disagree, somewhat agree, strongly agree.

- My healthcare professional is willing to discuss my medication concerns and is open to trying different treatments
- My healthcare professional understands the severity of my headache symptoms and the impact headaches have on my day-to-day life
- I worry that if I ask too many questions, my healthcare professional will think I'm a difficult patient
- I wish my healthcare provider and I talked more about my headache management goals
- I am comfortable seeking a new healthcare professional if I don't feel my current provider is meeting my needs
- I feel comfortable discussing my full range of symptoms with my healthcare professional
- I feel comfortable talking about access to treatment options based on my insurance coverage
- My healthcare professional spends enough time in our visits and effectively answers my questions

Which of the following, if any, do you wish your healthcare provider who currently manages your headaches, better understood about your experiences living with headaches? Please select all that apply.

- How headaches affect my mental/emotional health
- How much I rely on him/her to help me with my headaches
- The inconvenience of my medications
- The stress that headaches cause me and my loved ones
- How much headaches disrupt my life (e.g., social life, job performance, schooling, etc.)
- How exhausted I am
- The amount of pain I experience when I have a headache
- That pain isn't the only or worst headache symptom I experience
- That I would like to talk to others who have headaches
- That I want to know why I have headaches
- That my current treatment is not working well enough
- That I'm not exaggerating or faking
- That it is expensive to pay for all of the medical visits and medications to treat my headaches
- How afraid I am of a future with headaches
- Other
- None

MAIN SURVEY: TREATMENT

For the following questions, please assume that days with "headache" also refers to days with migraine and/or other types of headache.

Have you ever used, taken, or done any of the following to treat your headaches? Please select all that apply.

OTC

- Acetaminophen (e.g., Tylenol, Excedrin)
- Nonsteroidal anti-inflammatory (NSAIDs) (e.g., aspirin, ibuprofen, naproxen sodium, diclofenac)

ACUTE

- Barbiturates (e.g., Amytal, Butisol, Nembutal, Fiornal, Fiorecet)
- Dihydroergotamine (e.g., D.H.E. 45, Migranal)
- Diclofenac (e.g., Cambia, Flector, Solaraze)
- Ditan (e.g., Reyvow)
- Ergotamines (e.g., Cafergot, Ergomar)
- Gepants (e.g., Ubrelvy, Nurtec ODT)
- Opioids (e.g., Oxycodone, Vicodin)
- Triptans (e.g., Imitrex, Maxalt, Zomig, Axert, Relpax, Frova, Amerge)

PREVENTIVE PRESCRIPTION

- Anti-epileptic drugs (AEDs) (e.g., Topamax, Zonegram, Keppra)
- CGRP antagonists (e.g., Aimovig, Ajovy, Emgality, Vyepti)
- Botox

OTHER

- Bio-behavioral therapies (cognitive behavioral therapy, biofeedback, relaxation therapies, mindfulness-based therapies)
- Cannabidiol (CBD)
- Chiropractic, physical therapy, or occupational therapy

- Hormonal birth control or hormone replacement therapy
- Massage therapy
- Marijuana/cannabis (THC)
- Nerve blocks
- Neuromodulators or neurostimulators (e.g., Cefaly, sTMS mini, gammaCore)
- Oral or dental devices (to correct grinding, clenching, TMD, apnea, etc.)
- Oxygen
- Vitamins, herbs, or other over-the-counter supplements (e.g., magnesium, B2, ginger, CoQ10, green tea, ginseng)
- Other complementary, integrative, or alternative therapy (e.g., acupuncture/dry needling)
- Other
- None
- Decline to answer

Do you currently (or in the past few months) use, take, or do any of the following to treat your headaches? Please select all that apply.

OTC
- Acetaminophen (e.g., Tylenol, Excedrin)
- Nonsteroidal anti-inflammatory (NSAIDs) (e.g., aspirin, ibuprofen, naproxen sodium, diclofenac)

ACUTE
- Barbiturates (e.g., Amytal, Butisol, Nembutal, Fiornal, Fiorecet)
- Dihydroergotamine (e.g., D.H.E. 45, Migranal)
- Diclofenacs (e.g., Cambia, Flector, Solaraze)
- Ditan (e.g., Reyvow)
- Ergotamines (e.g., Cafergot, Ergomar)
- Gepants (e.g., Ubrelvy, Nurtec ODT)
- Opioids (e.g., Oxycodone, Vicodin)
- Triptans (e.g., Imitrex, Maxalt, Zomig, Axert, Relpax, Frova, Amerge)

PREVENTIVE PRESCRIPTION
- Antipileptic drugs (AEDs) (e.g., Topamax, Zonegram, Keppra)
- CGRP antagonists (e.g., Aimovig, Ajovy, Emgality, Vyepti)
- Botox

OTHER
- Bio-behavioral therapies (cognitive behavioral therapy, biofeedback, relaxation therapies, mindfulness-based therapies)
- Cannabidiol (CBD)
- Chiropractic, physical, or occupational therapy
- Hormonal birth control or hormone replacement therapy
- Massage therapy
- Marijuana/cannabis (THC)
- Nerve blocks
- Neuromodulators or neurostimulators (e.g., Cefaly, sTMS mini, gammaCore)
- Oral or dental devices (to correct grinding, clenching, TMD, apnea, etc.)
- Oxygen
- Vitamins, herbs, or other over-the-counter supplements (e.g., magnesium, B2, ginger, CoQ10, green tea, ginseng)
- Other complementary, integrative, or alternative therapy (e.g., acupuncture/dry needling)
- Other
- None
- Decline to answer

Please answer the following questions about the medication(s) that you currently use to treat headaches. Responses include: never, rarely, less than half the time, half the time or more

- Are you able to quickly return to your normal activities (i.e., work, family, leisure, social activities) after taking your headache medication?
- After taking your headache medication, are you pain free within 2 hours for most attacks?
- Does one dose of your headache medication usually relieve your headache and keep it away for at least 24 hours?
- Is your headache medication well tolerated?
- Are you comfortable enough with your headache medication to be able to plan your daily activities?
- After taking your headache medication, do you feel in control of your headaches enough so that you feel there will be no disruption to your daily activities?

You indicated that you are currently taking prescription medication(s) to help manage your headaches. Ideally, what would you most like to change, if anything, about your current headache medication(s)? Please select all that apply.

- Effectiveness in treating my most bothersome headache symptoms
- Effectiveness in helping me resume daily activities during/after a headache
- Number, severity, or duration of side effects
- How often I need to take it
- The cost or coverage available from my healthcare system/insurance
- The number of medications I need to take
- How long it takes for my medication to kick in
- How long the medication helps before it wears off
- Other
- There is nothing I would change about my current headache medication

For which of the following reasons, if any, have you paused or stopped headache medication recommended by your healthcare professional? Please select all that apply.

- I have not paused or stopped medication since I began
- Severity of side effects outweighed the effectiveness of medication
- How often I need to take medication(s)
- The cost or coverage available from my healthcare system/insurance
- The number of medications I need to take
- The medication wasn't effective or became significantly less effective over time
- The medication wasn't effective or became significantly less effective at relieving headache symptoms
- Tired of taking medications that didn't work
- Having to keep diaries and headache records
- Hassles with insurance companies
- Other

DEMOGRAPHICS

What is the highest level of education you have completed?

- Less than high school
- Completed some high school
- High school graduate
- Job-specific training program(s) after high school
- Some college, but no degree
- Associate degree
- Bachelor's degree (such as BA, BS)
- Some graduate school, but no degree
- Graduate degree (such as MBA, MS, MD, PhD)

How much total combined income did all members of your household earn before taxes last year?

This includes money from jobs; net income from business, farm, or rent; pensions; dividends; interest; social security payments; and any other money income received by members of your household who are eighteen (18) years of age or older.

- Less than $15,000
- $15,000 to $24,999
- $25,000 to $34,999
- $35,000 to $49,999
- $50,000 to $74,999
- $75,000 to $99,999
- $100,000 to $124,999
- $125,000 to $149,999
- $150,000 to $199,999
- $200,000 to $249,999
- $250,000 or more
- Prefer not to answer

What is your current marital status?

- Never married
- Married or civil union
- Divorced
- Separated
- Widowed
- Living with partner

Including yourself, how many people age 18 or older live in your household?

|__|__|

How many people under the age of 18 live in your household?

|__|__|

Which of the following best describes your employment status?

- Employed full time
- Employed part time
- Self-employed full time
- Self-employed part time
- Not employed, but looking for work
- Not employed and not looking for work
- Not employed, unable to work due to a disability or illness
- Retired
- Student
- Stay-at-home spouse or partner

Do you have any of the following types of health insurance? Please select all that apply.

- Health insurance provided by my employer
- Health insurance provided by a family member's employer
- Individual insurance policy bought by myself/a family member
- Health insurance from an exchange (i.e., marketplace)
- Medicare
- Medicaid
- Health insurance provided to students (e.g., through a college or university)
- Veterans benefits (e.g., active military, veterans, TriCare, reserve)
- Other
- None

Have you ever personally served in any branches of the U.S. Armed Forces (e.g., Army, Marine Corps, Navy, Air Force, National Guard, Coast Guard)?

- Yes, in the past, but not now
- Yes, currently
- No, never served in the U.S. Armed Forces

WEBOGRAPHICS

On how many different occasions did you do vigorous physical exercise during the past 30 days? [RANGE: 0-120]

I__I__I__I

On how many separate occasions would you say you watched news programs on TV during the past 30 days? [RANGE: 0-120]

I__I__I__I

Do you think that community service should be a requirement in schools?

- Yes
- No

Have you chosen not to purchase a product or service made by a particular company because the company's actions or policies conflicted with your values or beliefs?

- Yes, in the past year
- Yes, more than 1 year ago but within the past 2 years
- Yes, more than 2 years ago but within the past 3 years
- Yes, more than 3 years ago
- No, I have never done this

Most companies want to know about customers' interests and lifestyle choices so they can tailor their services and products to each customer's personal preferences. In general, do you see this as a good thing?

- Yes
- No
